# *Ureaplasma*-Driven Neonatal Neuroinflammation: Novel Insights from an Ovine Model

**DOI:** 10.1007/s10571-022-01213-8

**Published:** 2022-03-25

**Authors:** Christine Silwedel, Matthias C. Hütten, Christian P. Speer, Christoph Härtel, Axel Haarmann, Birgit Henrich, Maud P. M. Tijssen, Abdullah Ahmed Alnakhli, Owen B. Spiller, Nicolas Schlegel, Silvia Seidenspinner, Boris W. Kramer, Kirsten Glaser

**Affiliations:** 1 University Children’s Hospital, University of Wuerzburg, Josef-Schneider-Str. 2, 97080 Wuerzburg, Germany; 2 Department of Pediatrics, Faculty of Health, Medicine and Life Sciences, Maastricht University Medical Center, P. Debyelaan 25, 6229 HX Maastricht, Netherlands; 3 Department of Neurology, University of Wuerzburg, Josef-Schneider-Str. 11, 97080 Wuerzburg, Germany; 4 Institute of Medical Microbiology and Hospital Hygiene, University Clinic of Heinrich-Heine University Duesseldorf, Universitaetsstr. 1, 40225 Duesseldorf, Germany; 5 Department of Radiology and Nuclear Medicine, Faculty of Health, Medicine and Life Sciences, Maastricht University Medical Center, P. Debyelaan 25, 6229 HX Maastricht, Netherlands; 6 Division of Infection and Immunity, Cardiff University School of Medicine, Heath Park, Cardiff, CF14 4XN UK; 7 Department of Surgery I, University of Wuerzburg, Oberduerrbacherstr. 6, 97080 Wuerzburg, Germany; 8 Division of Neonatology, Department of Women’s and Children’s Health, Center for Pediatric Research Leipzig, University of Leipzig Medical Center, Liebigstraße 20a, 04103 Leipzig, Germany

**Keywords:** *Ureaplasma parvum*, CNS Integrity, Neonatal meningitis, Preterm birth, Immaturity, Animal model

## Abstract

*Ureaplasma* species (spp.) are considered commensals of the adult genitourinary tract, but have been associated with chorioamnionitis, preterm birth, and invasive infections in neonates, including meningitis. Data on mechanisms involved in *Ureaplasma*-driven neuroinflammation are scarce. The present study addressed brain inflammatory responses in preterm lambs exposed to *Ureaplasma parvum* (UP) in utero. 7 days after intra-amniotic injection of UP (*n* = 10) or saline (*n* = 11), lambs were surgically delivered at gestational day 128–129. Expression of inflammatory markers was assessed in different brain regions using qRT-PCR and in cerebrospinal fluid (CSF) by multiplex immunoassay. CSF was analyzed for UP presence using *ure*B-based real-time PCR, and MRI scans documented cerebral white matter area and cortical folding. Cerebral tissue levels of atypical chemokine receptor (ACKR) 3, caspases 1-like, 2, 7, and C–X–C chemokine receptor (CXCR) 4 mRNA, as well as CSF interleukin-8 protein concentrations were significantly increased in UP-exposed lambs. UP presence in CSF was confirmed in one animal. Cortical folding and white matter area did not differ among groups. The present study confirms a role of caspases and the transmembrane receptors ACKR3 and CXCR4 in *Ureaplasma*-driven neuroinflammation. Enhanced caspase 1-like, 2, and 7 expression may reflect cell death. Increased ACKR3 and CXCR4 expression has been associated with inflammatory central nervous system (CNS) diseases and impaired blood–brain barrier function. According to these data and previous in vitro findings from our group, we speculate that *Ureaplasma*-induced caspase and receptor responses affect CNS barrier properties and thus facilitate neuroinflammation.

## Background

Prematurity, particularly delivery at gestational ages < 30 weeks, remains the most important contributor to neonatal morbidity and mortality, thus constituting a major medical challenge (Liu et al. 2012; Stoll et al. 2015). Chorioamnionitis is one of the major risk factors for preterm birth (Ireland and Keelan 2014) and has been strongly related to ascending infection with *Ureaplasma species* (spp.) (Kasper et al. 2010; Goldenberg et al. 2000). As some of the smallest self-replicating bacteria, *Ureaplasma (U.) urealyticum* and *U. parvum* (UP) are common colonizers of the adult genitourinary tract (Waites et al. 2005). Although they are often regarded as low virulent, *Ureaplasma* spp. may evoke ascending infections in pregnant women (Waites et al. 2005). Consecutive amniotic invasion may lead to maternal and fetal inflammation, ultimately provoking preterm birth. In preterm and term neonates, *Ureaplasma* spp. may cause invasive infections, such as pneumonia and sepsis (Sweeney et al. 2017; Goldenberg et al. 2008; Silwedel et al. 2017; Viscardi 2014). In preterm neonates, *Ureaplasma* spp. have furthermore been associated with the development of chronic morbidities, such as bronchopulmonary dysplasia (BPD) (Silwedel et al. 2017; Viscardi 2014; Kasper et al. 2011; Groneck et al. 2001; Glaser et al. 2019). There is also culminating evidence linking *Ureaplasma* spp. to neonatal neuroinflammation and associated sequelae. *Ureaplasma* spp. were identified as causative pathogens in a relevant number of cases of neonatal meningitis, and some authors described an association between *Ureaplasma* spp. and intraventricular hemorrhage (IVH) or adverse neurodevelopmental outcome (Silwedel et al. 2017, 2020; Kasper et al. 2011; Viscardi et al. 2008; Glaser and Speer 2015; Berger et al. 2009; Rittenschober-Böhm et al. 2021). These observations are supported by in vitro data showing *Ureaplasma* spp. modulating brain immune defense mechanisms (Silwedel et al. 2018, [Bibr CR36][Bibr CR39], c).

Inflammation is orchestrated and carefully balanced by numerous mediators. Among these are pro-inflammatory cytokines, including tumor necrosis factor (TNF), interleukin (IL)-1β, IL-6, and interferons (IFN); cytokines bearing anti-inflammatory effects, such as IL-10 and IL-1 receptor antagonist (RA); the chemokines IL-8 and macrophage inflammatory proteins (MIP); as well as monocyte chemoattractant proteins (MCP) (Le Thuc et al. 2015). Adhesion molecules such as intercellular adhesion molecule (ICAM) 1 and vascular cell adhesion molecule (VCAM) 1 promote inflammatory tissue invasion (Wevers and Vries 2016), and growth factors like vascular endothelial growth factor (VEGF) or granulocyte colony-stimulating factor (G-CSF) facilitate vascular permeability and neutrophil inflammation, respectively (Wevers and Vries 2016; Hamilton 2008). Cell death appears to be closely associated with inflammation, with caspases acting as key mediators in inflammatory cell death as well as apoptosis (Cohen 1997; Shaalan et al. 2018). Furthermore, the blood–brain barrier (BBB) is highly relevant for neuroinflammation, physiologically shielding the brain from external injurious impacts (Williams et al. 2014). Several neuroinflammatory conditions are accompanied by BBB impairment, and mediators potentially involved include the transmembrane receptors atypical chemokine receptor (ACKR) 3 as well as C–X–C chemokine receptor (CXCR) 4, both permitting inflammatory cell migration into the central nervous system (CNS) (Williams et al. 2014; Huang et al. 2013; Moll et al. 2009).

To date, only few animal data are available on *Ureaplasma*-driven neuroinflammation, and the overall results are contradictory (Normann et al. 2009; Kelleher et al. 2017; Gussenhoven et al. 2017; Senthamaraikannan et al. 2016; Novy et al. 2009). Using an established preclinical animal model of *Ureaplasma*-mediated chorioamnionitis (Gussenhoven et al. 2017), the present study addressed brain inflammatory responses in preterm lambs after intrauterine UP exposure.

## Methods

### Animal Experiments

This study was performed with approval of the institutional Animal Ethics Research Committee of Maastricht University and the Dutch Central Animal Research Commission (CCD) (number AVD107002015225-2). As a comprehensive trial assessing the effects of prenatal UP exposure on different organ systems, the study was powered for the primary endpoint BPD, and sample size calculations were performed accordingly. Due to animal welfare regulations, the total number of animals included in the study was limited and, therefore, the study has not been powered for the secondary outcomes addressed in this manuscript.

Time-mated ewes were randomly assigned to one of two study groups (Table 1). At 121 or 122 days of gestation, animals received ultrasound-guided intra-amniotic injection of 5 × 10^5^ color changing units of UP serovar 3 (strain HPA5 (Rowlands et al. 2021), kindly provided by Prof. Dr. Owen B. Spiller) (UP group) or saline (control group). This concentration was shown to induce systemic organ inflammation in the ovine fetus (Ophelders et al. 2021). Lambs were delivered via cesarean section at day 128 or 129 (term ~ 150 days) and sacrificed by an intravenous injection of 1 g pentobarbital. Natural differences in breeding success were responsible for differing numbers of lambs in the UP group (*n* = 10) and the control group (*n* = 11). Due to hygienic reasons, blinding of the animal experiments was not possible, whereas data analysis was conducted blinded.

**Table 1:** Study animals and main characteristics

	Control	UP	*p*
*N* (total)	11	10	
Sex (m:f)	2:3 (*n* = 5^a^)	1:1 (*n* = 10^a^)	n.s
Gestational age (days)	128.6 ± 0.5 (*n* = 9^a^)	128.6 ± 0.5 (*n* = 10^a^)	n.s
Birth weight (g)	2508 ± 613 (*n* = 9^a^)	2364 ± 665 (*n* = 10^a^)	n.s
Brain weight (g)	35.3 ± 5.4 (*n* = 7^a^)	37.1 ± 5.6 g (*n* = 9^a^)	n.s
Brain tissue (PCR)	(*n* = 5^a^)	(*n* = 10^a^)	
Brain MRI	(*n* = 5^a^)	(*n* = 4^a^)	
CSF samples	(*n* = 5^a^)	(*n* = 5^a^)	

Animals did not significantly differ between control and UP group

^a^Data available for the given numbers of animals

### Sampling Protocol

Upon necropsy, body weight was determined, and cerebrospinal fluid (CSF) was collected by lumbar puncture immediately postmortem to be stored at − 80 °C. Brains were removed, weighted, and hemispheres were separated. The left hemisphere was dissected into different regions and snap frozen at − 80 °C. The right hemisphere was fixed using 4% paraformaldehyde solution (PFA, VWR Chemicals, Amsterdam, the Netherlands, cat. no. 11699408). After 3 months, PFA was replaced with phosphate-buffered saline (PBS, Gibco, Thermo Fisher Scientific, Waltham, MA, USA, cat. no. 11503387) containing 1% sodium azide (Merck, Kenilworth, NJ, USA, cat. no. 103692K).

### MRI Tissue Procedure and Brain Analysis

For magnetic resonance imaging (MRI), brain hemispheres were washed with PBS and placed in a closed vessel containing Fomblin solution (Sigma-Aldrich, St. Louis, MO, USA) to reduce artifacts and mimic in vivo brain surroundings. MR imaging was performed using a 3 Tesla MRI scanner (Achieva, Philips Healthcare, Best, the Netherlands) and a flex-M coil. Sagittal, axial, and coronal T2-weighted MRI sequences were used as well as axial inversion recovery T1-weighted sequences. Acquisition parameters were as follows: sagittal T2: field of view (FOV) 100 mm, slice thickness 1.8 mm, repetition time (RT) 3000 ms, echo time (ET) 90 ms, acquisition time (AT) 120,953 ms, and matrix 288 × 252; axial T2: FOV 120 mm, slice thickness 2 mm, RT 4000 ms, ET 90 ms, AT 120,512 ms, and matrix 300 × 242; coronal T2: FOV 100 mm, slice thickness 1.8 mm, RT 3000 ms, ET 90 ms, AT 121,539 ms, and matrix 312 × 271; and axial inversion recovery: FOV 100 mm, slice thickness 2 mm, RT 7000 ms, ET 15 ms, inversion time 600 ms, AT 122,136 ms, and matrix 200 × 154. Sagittal plane was used to determine cortical folding by calculation of the ratio between surface area and gyration, whereas white matter area in cm^2^ was measured in coronal plane. Syngo.via software (Siemens Healthineers, Erlangen, Germany) was employed for post-acquisition processing.

### Cytokine and Caspase Quantitative Real-Time Reverse Transcriptase Polymerase Chain Reaction (qRT-PCR)

Snap frozen tissue from brain frontal cortex (BFC) and brain periventricular zones (BPZ) was homogenized (BioMasherII Closed System Micro Tissue Homogenizer, Thermo Fisher Scientific, cat. no. 15344182). The NucleoSpin® RNA Kit (Macherey–Nagel, Dueren, Germany, cat. no. 740955.250) was employed to extract total RNA, which was eluted in 60 μL RNAse-free H_2_O (Macherey–Nagel) and stored at − 80 °C until reverse transcription. Total RNA was quantified (Qubit RNA BR Assay Kit, cat. no. Q10211, and Qubit® 2.0 Fluorometer, both Thermo Fisher Scientific), and 0.19–0.25 μg of total RNA was reverse transcribed using the High-Capacity cDNA Reverse Transcription Kit (Thermo Fisher Scientific, cat. no. 4368814). Following 1:10 dilution with nuclease-free H_2_O (Sigma-Aldrich, cat. no. W3513), cDNA was analyzed in duplicates of 25 μL reaction mixture containing 12.5 μL iTaq™ Universal SYBR® Green Supermix (Bio-Rad Laboratories, Hercules, CA, USA, cat. no. 172-5124), 0.5 μL nuclease-free H_2_O, and 1 μL each of a forward and reverse 10 μM primer solution (Sigma-Aldrich, Merck, Germany). Primer sequences are given in Table 2. Employing an Applied Biosystems® 7500 Real-Time PCR System (Thermo Fisher Scientific), the 2-step PCR protocol included an initial denaturation at 95 °C for 10 min and 40 cycles of 95 °C for 15 s and 60 °C for 1 min. Each run was concluded with a melt curve analysis confirming single PCR products. Amplification was normalized to the housekeeping gene peptidylprolyl isomerase C (PPIC, Sigma-Aldrich, Table 2). Mean fold changes in mRNA expression were determined with the help of the ΔΔC_T_ method (Livak and Schmittgen 2001).

**Table 2:** Ovine primers used for qRT-PCR

Name	Gene symbol	Sequence accession #	Orientation	Sequence [5′ to 3′]
PPIC	*PPIC*	XM_004008676.4	Forward	GCACATTTCATCGCGTCATCA
			Reverse	TGACCCACCCAATGCCATAA
CXCR4	*CXCR4*	NM_001277168.1	Forward	GGACTTGAGTAGCCGGTAGC
			Reverse	CGGAAGCAGGGTTCCTTCAT
IL-6	*IL6*	NM_001009392.1	Forward	ACCTGGACTTCCTCCAGAAC
			Reverse	TTGAGGACTGCATCTTCTCC
IL-8	*CXCL8*	NM_001009401.2	Forward	ATGAGTACAGAACTTCGA
			Reverse	TCATGGATCTTGCTTCTC
IL-10	*IL10*	NM_001009327.1	Forward	CCAGGATGGTGACTCGACTAGAC
			Reverse	TGGCTCTGCTCTCCCAGAAC
MCP-1	*MCP1*	XM_027956985.1	Forward	TGGGAAGCTCAATCAGCG
			Reverse	GCTGCAGTAACATGATGTCG
MCP-3	*MCP3*	NM_001009411.2	Forward	CACCATCACGGACCAAGAGAG
			Reverse	ATCCGTCATCTCAGCCTTCC
TNF	*TNF*	NM_001024860.1	Forward	CTGGGCAGGTCTACTTTGGG
			Reverse	GAAGGGGATGAGGAGGGTCT
VEGF	*VEGFA*	NM_001025110.1	Forward	TTGCCTTGCTGCTCTACCTT
			Reverse	GGGCACACACTCCAGACTTT
ACKR3	*ACKR3*	XM_004001768.3	Forward	CGGTCTGGGATACGGAACAA
			Reverse	GCCGTGTTACAGACTGGGAT
G-CSF	*CSF3*	XM_027975456.1	Forward	TGCGCTATAGACGCCATGAG
			Reverse	CCATGTTCCCAGTCTCACCC
IL-1RA	*IL1RN*	NM_001308595.1	Forward	AGATAGATGTGGTACCCATCG
			Reverse	TTCACAGCCTCTAACTTGAGC
ICAM-1	*ICAM1*	XM_027969187.1	Forward	TATGTCCTGCCATCGACCG
			Reverse	ACATAGACCTCAGCGTCCG
VCAM-1	LOC101113636	XM_004002233	Forward	GGTGAAGCTCTACTCCTTCC
			Reverse	AAACAATTCAATCTCCAGCCG
Caspase 1-like	LOC101117013	XM_004015962.4	Forward	CTCACTTCAGGTTCACAGTC
			Reverse	TATTCTTTGGGCTGTTTCTGG
Caspase 2	*CASP2*	XM_012177298.3	Forward	CTGCCGTGGAGATGAAACAG
			Reverse	GCGTAGCCACAAATCATGTC
Caspase 3	*CASP3*	XM_015104559.2	Forward	AAATGCAACTCTTCCACCAG
			Reverse	TGTTTCTTCCTCCTACCTCAC
Caspase 7	*CASP7*	XM_012102956.3	Forward	AAACCCTGTTAGAGAAGCCC
			Reverse	TGAATAATAGCCTGGAACTGTG
Caspase 9	*CASP9*	XM_012187488	Forward	GATGTCCTGTGTCCGTTGAG
			Reverse	GTCTTTCTGCTCTCCACCAC
Caspase 14	*CASP14*	XM_004008465.4	Forward	GCCCTTTCTCCAAGGTCAG
			Reverse	TGTCGTATGTCTCCTCTTCC

### CSF *Ureaplasma* qPCR

CSF samples were assessed for DNA of UP at the Institute of Medical Microbiology and Hospital Hygiene, Duesseldorf, Germany, using *ure*B-specific primers (UP-F: AGGAAATGAAGATAAAGAACGCAAA and UP-R: AACGAATAGCAGTACCTGATGGAAT) and probe (UP-S: HEX-TTGCTTATGGACGACGTTTCG-BHQ1) and a qPCR protocol described previously (Mobius et al. 2012). UP serovar 3 (strain HPA5) was included as a positive control.

### Multi-analyte Immunoassay

CSF concentrations of pro- and anti-inflammatory mediators were determined by means of bead-based immunoassay using Luminex® reagent kits (Merck Millipore, Merck group, Darmstadt, Germany, cat. no. BCYT1-33 K-PX15). Lower detection limits were 0.05 pg/mL (IFN-γ), 0.02 pg/mL (IL-1α), 0.71 pg/mL (IL-1β), 1.81 pg/mL (IL-4), 1.68 pg/mL (IL-6), 5.6 pg/mL (IL-8), 0.12 pg/mL (IL-10), 0.06 pg/mL (IL-17A), 0.0 pg/mL (IL-36 RA), 1.82 pg/mL (IFN-γ-induced protein (IP) 10), 2.89 pg/mL (MCP-1), 8.39 pg/mL (MIP-1α), 3.11 pg/mL (MIP-1β), 2.01 pg/mL (TNF), and 0.52 pg/mL (VEGF); values underneath were set to 0. A standard curve was aligned using xPonent® Software (Luminex Cooperation, Austin, TX, USA), and cytokine concentrations were calculated from this curve. Samples were analyzed in duplicate.

### Statistical Analysis

Results were analyzed using GraphPad Prism software (version 6.01, GraphPad Software, San Diego, CA, USA). Non-parametric Mann–Whitney *U* test was employed for assessment of differences among groups. Data were expressed as means ± standard deviation (SD), and results at *p* < 0.05 were considered significant.

## Results

### Study Population and Animal Characteristics

Animals assigned to the two study groups did not significantly differ in sex, gestational age, and birth weight (Table 1). No significant differences in brain weight were observed between UP exposed and control animals (Table 1).

### Brain MRI

**Fig. 1:**
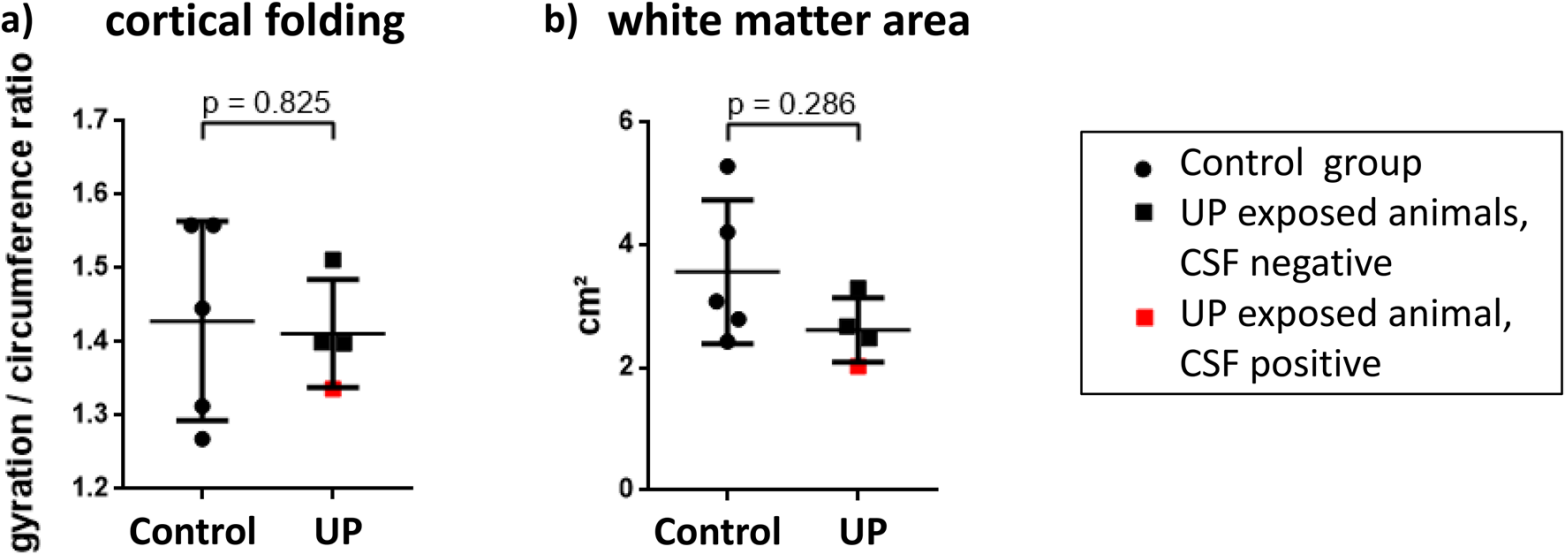
MRI scans were used to assess a potential influence of prenatal *U. parvum* exposure on cortical folding (**a**, sagittal plane) and brain white matter area (**b**, coronal plane). Results are presented in scatter plots showing means ± SD, comparing the control group (*n* = 5) and the group exposed to *U. parvum* (UP, *n* = 4)**.** The animal with a positive CSF *Ureaplasma* PCR is marked in red

Apart from minor intraventricular air due to the ex vivo experiment, no macroscopic abnormalities were detected. Cortical folding and white matter area did not differ significantly between the UP and the control group (Fig. 1).

### Tissue Inflammation Markers

**Fig. 2:**
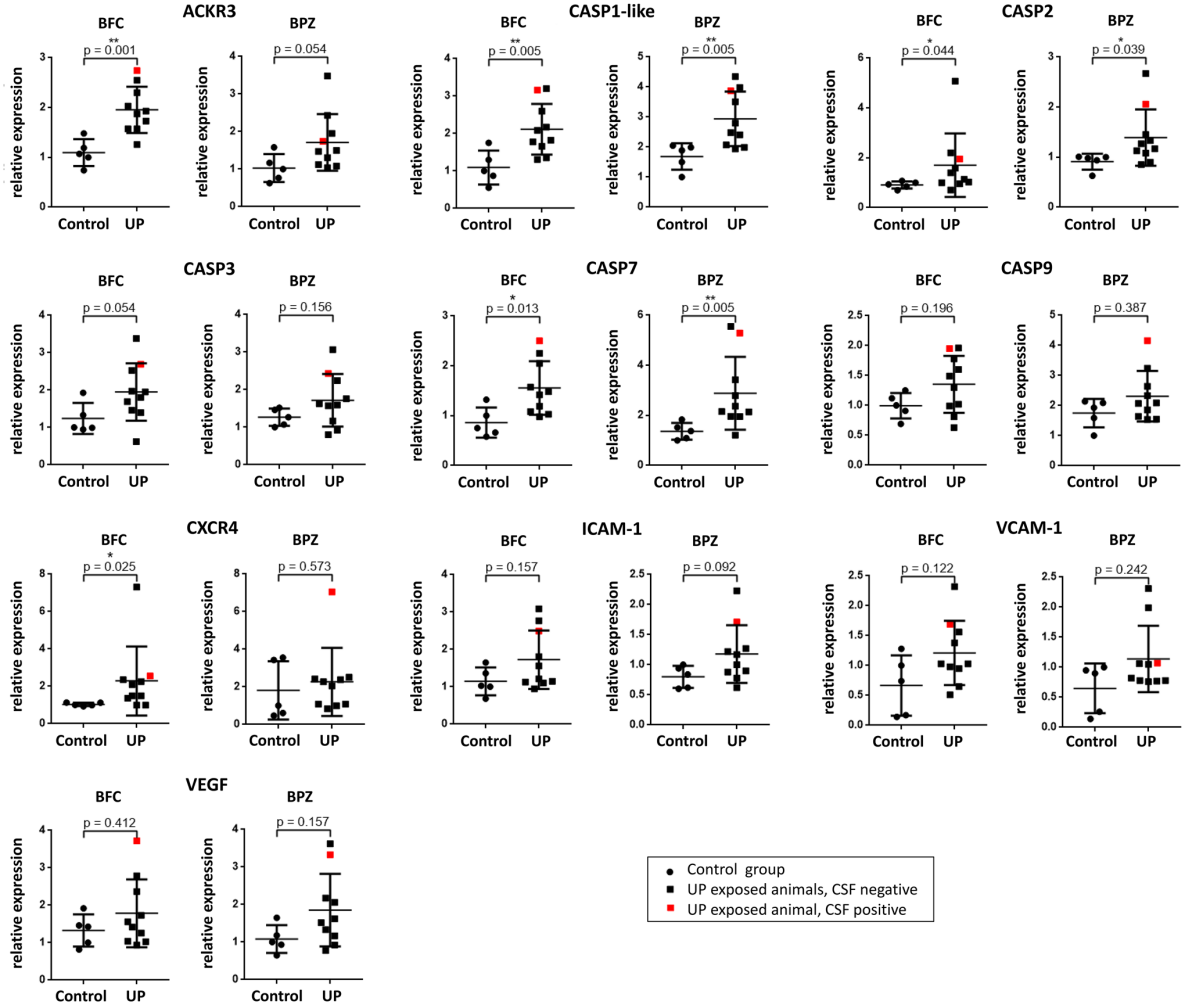
Brain tissue mRNA expression of ACKR3, caspase (CASP) 1-like, CASP2, CASP3, CASP7, CASP9, CXCR 4, ICAM-1, VCAM-1, and VEGF was assessed for BFC and BPZ. Scatter plots present individual data points as well as means ± SD. *U. parvum*-exposed animals (UP, *n* = 10) were compared to control animals (*n* = 5). The animal tested positive for UP is marked in red. *p < 0.05, **p < 0.01 vs. control

ACKR3 mRNA expression was found to be significantly elevated in the UP group (BFC: 1.78-fold ± 0.42, Mann–Whitney *U* test*, U* = 1.000, *p* = 0.001, vs. control animals, Fig. 2). Moreover, Mann–Whitney *U* tests revealed significant differences for caspase 1-like mRNA (BFC: 1.93-fold ± 0.62, *U* = 3.000, *p* = 0.005; BPZ: 1.74-fold ± 0.54, *U* = 3.500, *p* = 0.005, vs. control animals), caspase 2 mRNA (BFC: 1.87-fold ± 1.40, *U* = 8.500, *p* = 0.044; BPZ: 1.52-fold ± 0.62, *U* = 8.000, *p* = 0.039), caspase 7 mRNA (BFC: 1.80-fold ± 0.62, *U* = 5.000, *p* = 0.013; BPZ: 2.12-fold ± 1.07, *U* = 3.000, *p* = 0.005), and CXCR4 mRNA (BFC: 2.21-fold ± 1.79, *U* = 7.000, *p* = 0.025) (Fig. 2). Caspase 3, caspase 9, ICAM-1, VCAM-1, and VEGF mRNA levels did not differ between both groups (Fig. 2). Caspase 14, G-CSF, IL-1RA, IL-6, IL-8, IL-10, MCP-1, MCP-3, and TNF were weakly or not expressed in either group (data not shown). Comparing frontal cortex tissue and tissue from the periventricular zone, no differences were detected (Fig. 2).

### CSF Cytokine Protein Expression

**Fig. 3:**
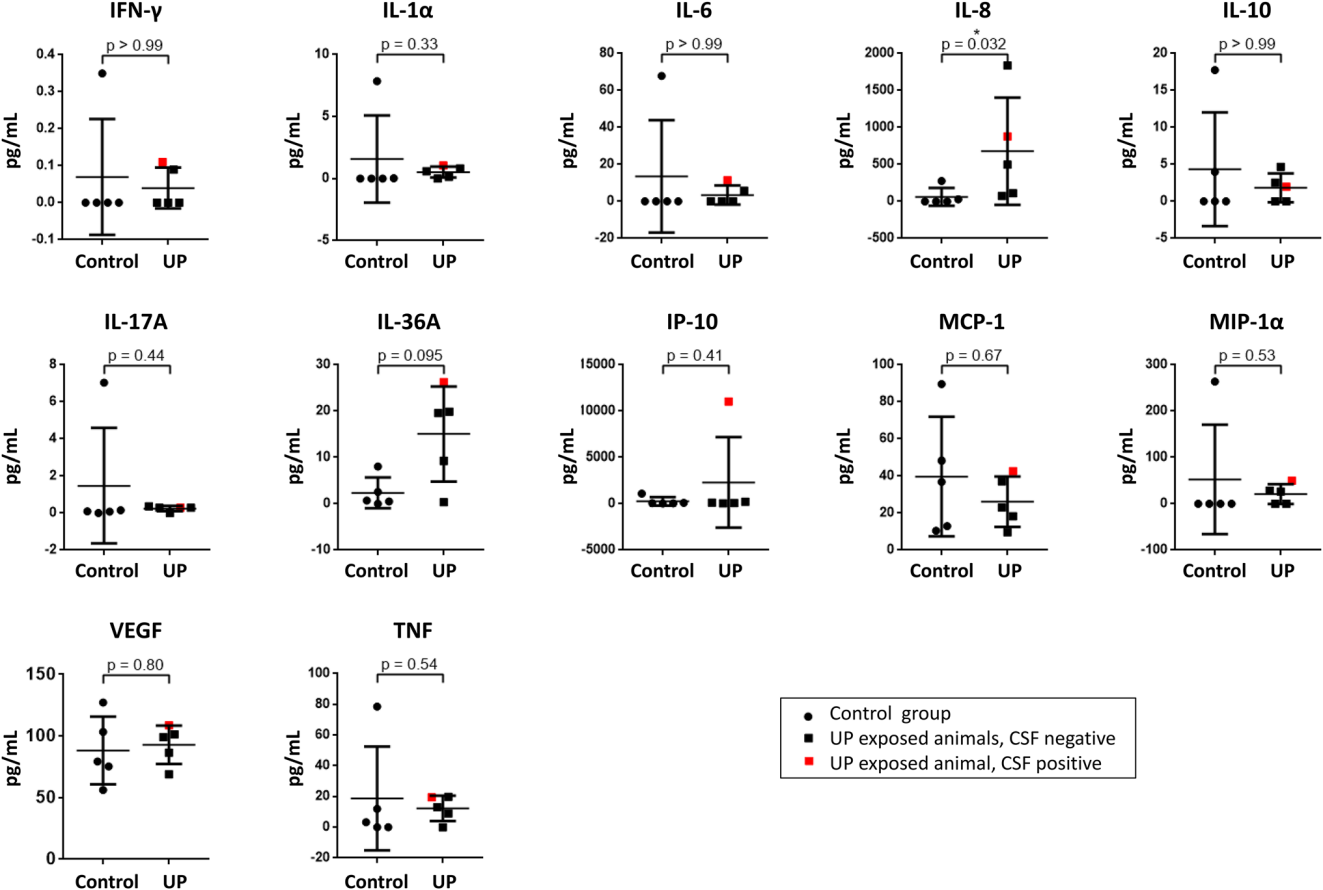
CSF protein concentrations of IFN-γ, IL-1α, IL-6, IL-8, IL-10, IL-17A, IL-36 RA, IP-10, MCP-1, MIP-1α, TNF, and VEGF depict responses to *Ureaplasma* exposure of fetal lambs (UP, *n* = 5) compared to control animals (*n* = 5). The CSF *Ureaplasma*-positive animal is marked in red. Data are shown as means ± SD, *p < 0.05 vs. control

Analysis of CSF cytokine levels showed a significant increase of IL-8 protein in UP-exposed animals (11.2 ± 11.9-fold, Mann–Whitney *U* test, *U* = 2.000, *p* = 0.032 vs. control, Fig. 3). No significant differences among both study groups were observed for IFN-γ, IL-1α, IL-6, IL-10, IL-17A, IL-36 RA, IP-10, MCP-1, MIP-1α, TNF, and VEGF (Fig. 3). IL-1β, IL-4, and MIP-1β protein were undetectable in either group.

### Detection of *Ureaplasma* spp. in CSF Samples

While all CSF samples of the control group remained PCR negative, UP DNA of the reference strain HPA5 was detected in 1 out of 5 samples of the UP group (1.63 × 10^4^ copy numbers / mL CSF).

### Singular Case: *Ureaplasma* CNS Invasion

**Fig. 4:**
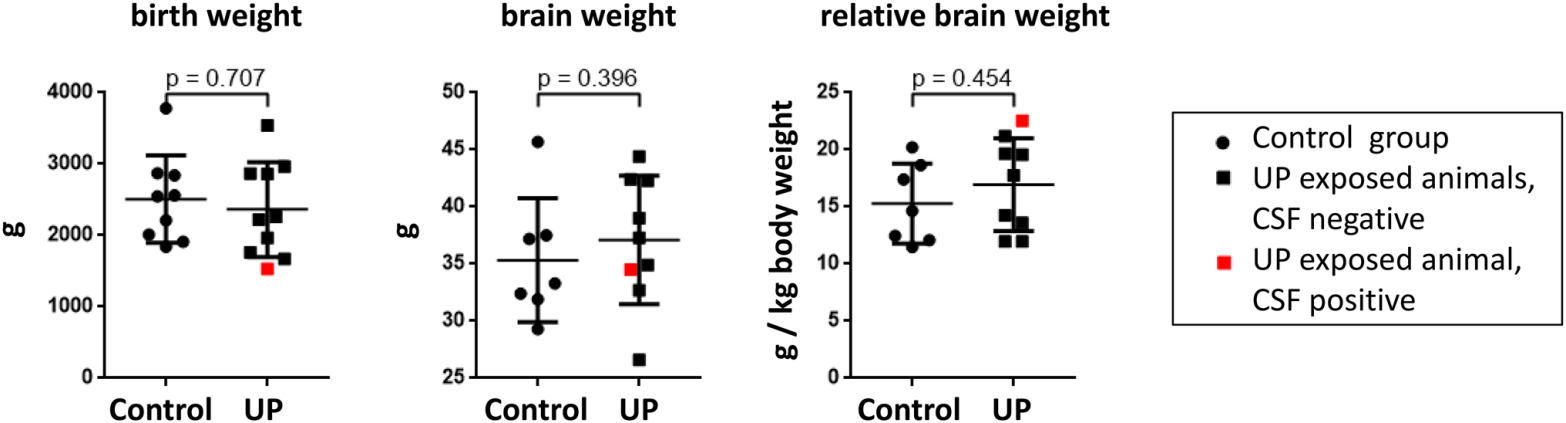
Scatter plots present somatic parameters itemized for the individual animals as well as means ± SD (please refer to Table 1 for *n*). The single animal with a positive CSF *Ureaplasma* PCR is marked in red

The one animal with proven UP invasion into the CSF distinguished itself from the rest of the study group in several categories (Figs. 1–4). With a birth weight below average, the animal’s relative brain weight was, vice versa, increased (Fig. 4). Cortical folding and white matter area were below average (Fig. 1). CSF IL-36A and IP-10 protein concentrations were distinctly higher than in all other animals (Fig. 3). Brain tissue mRNA levels were increased for ACKR3, caspase 1-like, caspase 2, caspase 7, caspase 9, CXCR4, ICAM-1, and VEGF (Fig. 2).

## Discussion

Prenatal, perinatal, and postnatal *Ureaplasma* exposure have been associated with neurological morbidities particularly in preterm infants, including meningitis, IVH, and adverse neurodevelopmental outcome (Silwedel et al. 2017, 2020; Kasper et al. 2011; Viscardi et al. 2008; Glaser and Speer 2015; Berger et al. 2009). So far, data on *Ureaplasma*-driven neuroinflammation are scarce, and current knowledge is based on single animal and few in vitro studies (Silwedel et al. 2020). This is the first study addressing inflammatory brain responses to acute intrauterine UP exposure in preterm sheep. Our results confirm a particular role of receptors regulating CNS barrier function as well as cell death-related caspases in *Ureaplasma*-driven neuroinflammation. The present data support the hypothesis that *Ureaplasma* infection affects CNS integrity (Silwedel et al. 2019a, b, c, 2018). Finally, our results demonstrate that *Ureaplasma* spp. are able to cross the BBB and enter the CNS.

Inflammation is a host defense mechanism triggered by infectious or non-infectious stimuli. A complex interplay of pro- and anti-inflammatory mediators is aimed at pathogen elimination, confining, at the same time, associated tissue injury (Le Thuc et al. 2015; Wevers and Vries 2016; Hamilton 2008). Apart from elevated CSF levels of IL-8, we did not detect a significant induction of classic pro- and anti-inflammatory mediators in response to UP exposure in this study (Figs. 2, 3). These findings are in line with previous clinical and in vitro studies. Whereas pronounced pro-inflammation was described in the airways and blood upon *Ureaplasma* infection, CSF invasion by *Ureaplasma* spp. did not evoke inflammatory cytokine responses in neonates and, similarly, in vitro studies did not reveal cytokine responses in *Ureaplasma*-stimulated human brain microvascular endothelial cells (HBMEC) (Glaser et al. 2019; Viscardi et al. 2008, 2006, 2002; Silwedel et al. 2019b, c; Glaser et al. 2018a, b; Glaser et al. 2017). These findings may be attributable to both an immune privileged state of the CNS and the pathogen itself. Either way, attenuated local cytokine responses upon *Ureaplasma* CNS infection may impede bacterial elimination and, ultimately, facilitate chronic infection and long-term neuroinflammation (Silwedel et al. 2020; Forrester et al. 2018). Notably, cases of chronic *Ureaplasma* meningitis with a history as long as 8 months have repetitively been described (Glaser and Speer 2015; Glaser et al. 2015).

Inflammation appears to be closely interlinked with programmed cell death (Shaalan et al. 2018). Caspases act as key agents both in inflammatory cell death as well as in apoptosis, with caspase 1 mainly mediating the former and caspases 2, 3, 7, and 9 being primarily involved in the latter (Cohen 1997; Man and Kanneganti 2016; Jorgensen et al. 2017). Our data revealed significantly enhanced brain mRNA levels of caspases 1-like, 2, and 7 in UP-exposed fetal lambs, as well as an increase in caspase 3 mRNA of borderline significance (Fig. 2). We furthermore observed *Ureaplasma*-induced increases in mRNA levels of the BBB receptors ACKR3 and CXCR4. These results are in accordance with previous in vitro data published by our group demonstrating *Ureaplasma*-driven cell death in HBMEC mediated by caspases as well as an induction of ACKR3 and CXCR4 in *Ureaplasma*-stimulated HBMEC (Silwedel et al. 2018, 2019a, c). Enhanced expression of these receptors has been recognized to promote inflammatory cell migration into the CNS and both have been associated with inflammatory CNS diseases (Moll et al. 2009; Liu and Dorovini-Zis 2009; Cruz-Orengo et al. 2011). Cell death, on the other hand, is intrinsically intended to eliminate particularly intracellular pathogens (Jorgensen et al. 2017). However, cell death in cells exerting physiological barrier and / or immune function may inadvertently facilitate tissue invasion by immune cells as well as pathogens. Since the present experimental setting did not allow functional assays, we cannot ultimately prove the impact of *Ureaplasma*-driven interferences with caspases and transmembrane receptors on in vivo brain barrier function. However, previous in vitro data confirmed reduced barrier properties in *Ureaplasma*-exposed HBMEC (Silwedel et al. 2019a). We hypothesize that induction of apoptosis-related caspases and up-regulation of receptors regulating passage into the CNS may impair CNS barrier properties and brain integrity.

In this study, prenatal UP exposure resulted in invasive CNS infection in one of the lambs, demonstrating the ability of UP to cross the BBB and invade the CNS. Closer assessment showed some interesting features in the respective animal, including the lowest birth weight within the cohort (Fig. 4). In neonates, *Ureaplasma* detection in cord blood has been associated with a significantly lower birth weight (Goldenberg et al. 2008). Vice versa, the CSF-positive animal held the highest relative brain weight (Fig. 4), possibly indicating brain edema as a reaction to invasive *Ureaplasma* CNS infection. MRI revealed cortical folding and white matter area below average in this animal (Fig. 1). These findings may be in line with previous animal studies showing structural changes upon prenatal *Ureaplasma* exposure (Normann et al. 2009; Kelleher et al. 2017). Furthermore, we observed pronounced caspase, ACKR3 and CXCR4 expression in this lamb’s brain tissue (Fig. 2), whereas only isolated CSF cytokines were increased (Fig. 3). The latter is in accordance with a clinical study in neonates, documenting no significant elevation of inflammatory cytokines in infants with CSF invasion by *Ureaplasma* spp. (Viscardi et al. 2008). Interestingly, however, IP-10 (syn. C–X–C chemokine ligand 10) was one of the mediators most pronounced in this animal’s CSF (Fig. 3). IP-10 has been ascribed a role in BBB disruption in neurodegenerative morbidities as well as in infectious diseases, emphasizing a potential role of barrier impairment in *Ureaplasma*-driven neuroinflammation (Wang et al. 2018; Ramesh et al. 2013). It remains to be determined if the presence of *Ureaplasma* in the CSF induced the exaggerated caspase and receptor response or, vice versa, if these reactions allowed passage of *Ureaplasma* into the CNS in the first place. Only two previous studies in rhesus macaques reported *Ureaplasma* CNS invasion upon *Ureaplasma* chorioamnionitis (Senthamaraikannan et al. 2016; Novy et al. 2009).

The few previous animal studies addressing *Ureaplasma*-driven neuroinflammation reported ambiguous results (Normann et al. 2009; Kelleher et al. 2017; Gussenhoven et al. 2017; Senthamaraikannan et al. 2016). In line with our own data, no evidence for brain inflammation, i.e., no cellular or cytokine responses in terms of classic pro- and anti-inflammatory mediators, was found upon acute intrauterine *Ureaplasma* infection in rhesus macaques (Senthamaraikannan et al. 2016). Whereas chronic prenatal *Ureaplasma* exposure was associated with abnormal brain development and cellular alterations in a macaque and ovine model, respectively (Kelleher et al. 2017; Gussenhoven et al. 2017), we did not detect consistent structural abnormalities upon intrauterine UP exposure in our study (Fig. 1). Timing and duration of prenatal *Ureaplasma* infection might be an important contributor determining clinical manifestation and potentially long-term outcome. A limitation of the present study was therefore the single time point of assessment. A longer duration of exposure reflecting chronic infection may have had induced different results. Furthermore, this study, like most animal studies, was limited by rather low numbers of animals within each group. Further in vivo and in vitro studies are essential to gain a full understanding of the impact of prenatal, perinatal, and postnatal *Ureaplasma* exposure in preterm infants and to gain better understanding of underlying mechanisms of *Ureaplasma* CNS infection.

## Conclusion

This is the first ovine study addressing preterm brain inflammatory responses upon acute intrauterine *Ureaplasma* infection. In line with previous in vitro data from our group, the current results depict that interference with BBB receptors and caspases rather than classic pro-inflammation appears to be the major mechanism in *Ureaplasma*-driven neuroinflammation. By increasing ACKR3 and CXCR4 expression, *Ureaplasma* spp. may impair CNS barrier function, while induction of caspases may induce cell death and tissue damage. Absent or mitigated local inflammatory responses could hamper pathogen eradication. In susceptible infants, ultimate consequence may be chronic infection and sustained neuroinflammation with subsequent long-term sequelae, as seen in clinical cases of *Ureaplasma* meningitis in preterm and term neonates.

## Data Availability

The datasets used and analyzed in the present study are available from the corresponding author on reasonable request.

## Abbreviations

ACKR: Atypical chemokine receptor
AT: Acquisition time
BBB: Blood–brain barrier
BFC: Brain frontal cortex
BPD: Bronchopulmonary dysplasia
BPZ: Brain periventricular zone
CNS: Central nervous system
CXCR: C–X–C chemokine receptor
ET: Echo time
FCS: Fetal calf serum
FOV: Field of view
G-CSF: Granulocyte colony-stimulating factor
HBMEC: Human brain microvascular endothelial cells
ICAM: Intercellular adhesion molecule
IFN: Interferon
IL: Interleukin
IVH: Intraventricular hemorrhage
IP: Interferon gamma-induced protein
MCP: Monocyte chemoattractant protein
MIP: Macrophage inflammatory protein
PBS: Phosphate-buffered saline
PFA: Paraformaldehyde
PPIC: Peptidylprolyl isomerase C
qRT-PCR: Real-time quantitative reverse transcriptase polymerase chain reaction
RA: Receptor antagonist
RT: Repetition time
SA: Sodium azide
SD: Standard deviation
spp.: Species
TNF: Tumor necrosis factor
U.: *Ureaplasma*
UP: *Ureaplasma parvum* Group
VEGF: Vascular endothelial growth factor
VCAM-1: Vascular cell adhesion molecule 1

## References

[CR1] Berger A, Witt A, Haiden N, Kaider A, Klebermasz K, Fuiko R (2009). Intrauterine infection with Ureaplasma species is associated with adverse neuromotor outcome at 1 and 2 years adjusted age in preterm infants. J Perinat Med.

[CR2] Cohen GM (1997). Caspases: the executioners of apoptosis. Biochem J.

[CR3] Cruz-Orengo L, Holman DW, Dorsey D, Zhou L, Zhang P, Wright M (2011). CXCR7 influences leukocyte entry into the CNS parenchyma by controlling abluminal CXCL12 abundance during autoimmunity. J Exp Med.

[CR4] Forrester JV, McMenamin PG, Dando SJ (2018). CNS infection and immune privilege. Nat Rev Neurosci.

[CR9] Glaser K, Speer CP (2015). Neonatal CNS infection and inflammation caused by Ureaplasma species: rare or relevant?. Expert Rev Anti Infect Ther..

[CR10] Glaser K, Wohlleben M, Speer CP (2015). An 8-month history of meningitis in an extremely low birth weight infant?—long-lasting Infection with *Ureaplasma parvum*. Z Geburtshilfe Neonatol.

[CR7] Glaser K, Silwedel C, Fehrholz M, Waaga-Gasser AM, Henrich B, Claus H (2017). Ureaplasma species differentially modulate pro- and anti-inflammatory cytokine responses in newborn and adult human monocytes pushing the state toward pro-inflammation. Front Cell Infect Microbiol..

[CR6] Glaser K, Silwedel C, Fehrholz M, Henrich B, Waaga-Gasser AM, Claus H (2018). Ureaplasma isolates stimulate pro-inflammatory CC chemokines and matrix metalloproteinase-9 in neonatal and adult monocytes. PLoS One..

[CR8] Glaser K, Silwedel C, Waaga-Gasser AM, Henrich B, Fehrholz M, Claus H (2018). Ureaplasma isolates differentially modulate growth factors and cell adhesion molecules in human neonatal and adult monocytes. Cytokine.

[CR5] Glaser K, Gradzka-Luczewska A, Szymankiewicz-Breborowicz M, Kawczynska-Leda N, Henrich B, Waaga-Gasser AM (2019). Perinatal Ureaplasma exposure is associated with increased risk of late onset sepsis and imbalanced inflammation in preterm infants and may add to lung injury. Front Cell Infect Microbiol..

[CR12] Goldenberg RL, Hauth JC, Andrews WW (2000). Intrauterine infection and preterm delivery. N Engl J Med.

[CR11] Goldenberg RL, Andrews WW, Goepfert AR, Faye-Petersen O, Cliver SP, Carlo WA (2008). The Alabama Preterm Birth Study: umbilical cord blood Ureaplasma urealyticum and Mycoplasma hominis cultures in very preterm newborn infants. Am J Obstet Gynecol..

[CR13] Groneck P, Schmale J, Soditt V, Stutzer H, Gotze-Speer B, Speer CP (2001). Bronchoalveolar inflammation following airway infection in preterm infants with chronic lung disease. Pediatr Pulmonol.

[CR14] Gussenhoven R, Ophelders D, Kemp MW, Payne MS, Spiller OB, Beeton ML (2017). The paradoxical effects of chronic intra-amniotic *Ureaplasma parvum* exposure on ovine fetal brain development. Dev Neurosci.

[CR15] Hamilton JA (2008). Colony-stimulating factors in inflammation and autoimmunity. Nat Rev Immunol.

[CR16] Huang J, Li Y, Tang Y, Tang G, Yang GY, Wang Y (2013). CXCR4 antagonist AMD3100 protects blood-brain barrier integrity and reduces inflammatory response after focal ischemia in mice. Stroke.

[CR17] Ireland DJ, Keelan JA (2014). The maternal serological response to intrauterine Ureaplasma sp. infection and prediction of risk of pre-term birth. Front Immunol.

[CR18] Jorgensen I, Rayamajhi M, Miao EA (2017). Programmed cell death as a defence against infection. Nat Rev Immunol.

[CR20] Kasper DC, Mechtler TP, Reischer GH, Witt A, Langgartner M, Pollak A (2010). The bacterial load of Ureaplasma parvum in amniotic fluid is correlated with an increased intrauterine inflammatory response. Diagn Microbiol Infect Dis.

[CR19] Kasper DC, Mechtler TP, Bohm J, Petricevic L, Gleiss A, Spergser J (2011). In utero exposure to Ureaplasma spp. is associated with increased rate of bronchopulmonary dysplasia and intraventricular hemorrhage in preterm infants. J Perinat Med..

[CR21] Kelleher MA, Liu Z, Wang X, Kroenke CD, Houser LA, Dozier BL (2017). Beyond the uterine environment: a nonhuman primate model to investigate maternal-fetal and neonatal outcomes following chronic intrauterine infection. Pediatr Res.

[CR44] Le Thuc O, Blondeau N, Nahon JL, Rovere C (2015). The complex contribution of chemokines to neuroinflammation: switching from beneficial to detrimental effects. Ann N Y Acad Sci.

[CR22] Liu KK, Dorovini-Zis K (2009). Regulation of CXCL12 and CXCR4 expression by human brain endothelial cells and their role in CD4+ and CD8+ T cell adhesion and transendothelial migration. J Neuroimmunol.

[CR23] Liu L, Johnson HL, Cousens S, Perin J, Scott S, Lawn JE (2012). Global, regional, and national causes of child mortality: an updated systematic analysis for 2010 with time trends since 2000. Lancet.

[CR24] Livak KJ, Schmittgen TD (2001). Analysis of relative gene expression data using real-time quantitative PCR and the 2(-Delta Delta C(T)) Method. Methods.

[CR25] Man SM, Kanneganti TD (2016). Converging roles of caspases in inflammasome activation, cell death and innate immunity. Nat Rev Immunol.

[CR26] Mobius N, Brenneisen W, Schaeffer A, Henrich B (2012). Protocol for the rapid detection of the urogenital tract mollicutes and Chlamydia with concomitant LGV-(sub)typing. Methods Mol Biol.

[CR27] Moll NM, Cossoy MB, Fisher E, Staugaitis SM, Tucky BH, Rietsch AM (2009). Imaging correlates of leukocyte accumulation and CXCR4/CXCL12 in multiple sclerosis. Arch Neurol.

[CR28] Normann E, Lacaze-Masmonteil T, Eaton F, Schwendimann L, Gressens P, Thebaud B (2009). A novel mouse model of Ureaplasma-induced perinatal inflammation: effects on lung and brain injury. Pediatr Res.

[CR29] Novy MJ, Duffy L, Axthelm MK, Sadowsky DW, Witkin SS, Gravett MG (2009). Ureaplasma parvum or *Mycoplasma hominis* as sole pathogens cause chorioamnionitis, preterm delivery, and fetal pneumonia in rhesus macaques. Reprod Sci.

[CR30] Ophelders D, Boots AW, Hütten MC, Al-Nasiry S, Jellema RK, Spiller OB (2021). Screening of Chorioamnionitis Using Volatile Organic Compound Detection in Exhaled Breath: A Pre-clinical Proof of Concept Study. Front Pediatr..

[CR31] Ramesh G, MacLean AG, Philipp MT (2013). Cytokines and chemokines at the crossroads of neuroinflammation, neurodegeneration, and neuropathic pain. Mediators Inflamm..

[CR32] Rittenschober-Böhm J, Habermüller T, Waldhoer T, Fuiko R, Schulz SM, Pimpel B (2021). Maternal vaginal Ureaplasma spp. colonization in early pregnancy is associated with adverse short- and long-term outcome of very preterm infants. Children (Basel).

[CR33] Rowlands RS, Kragh K, Sahu S, Maddocks SE, Bolhuis A, Spiller OB (2021). A requirement for flow to enable the development of Ureaplasma parvum biofilms in vitro. J Appl Microbiol..

[CR34] Senthamaraikannan P, Presicce P, Rueda CM, Maneenil G, Schmidt AF, Miller LA (2016). Intra-amniotic *Ureaplasma parvu*m-induced maternal and fetal inflammation and immune responses in rhesus macaques. J Infect Dis.

[CR35] Shaalan A, Carpenter G, Proctor G (2018). Caspases are key regulators of inflammatory and innate immune responses mediated by TLR3 in vivo. Mol Immunol.

[CR37] Silwedel C, Speer CP, Glaser K (2017). Ureaplasma-associated prenatal, perinatal, and neonatal morbidities. Expert Rev Clin Immunol.

[CR38] Silwedel C, Speer CP, Haarmann A, Fehrholz M, Claus H, Buttmann M (2018). Novel insights into neuroinflammation: bacterial lipopolysaccharide, tumor necrosis factor alpha, and Ureaplasma species differentially modulate atypical chemokine receptor 3 responses in human brain microvascular endothelial cells. J Neuroinflamm.

[CR36] Silwedel C, Haarmann A, Fehrholz M, Claus H, Speer CP, Glaser K (2019). More than just inflammation: Ureaplasma species induce apoptosis in human brain microvascular endothelial cells. J Neuroinflamm.

[CR39] Silwedel C, Speer CP, Haarmann A, Fehrholz M, Claus H, Schlegel N (2019). Ureaplasma species modulate cell adhesion molecules and growth factors in human brain microvascular endothelial cells. Cytokine..

[CR40] Silwedel C, Speer CP, Haarmann A, Fehrholz M, Claus H, Schlegel N (2019). Ureaplasma species modulate cytokine and chemokine responses in human brain microvascular endothelial cells. International journal of molecular sciences..

[CR41] Silwedel C, Speer CP, Härtel C, Glaser K (2020). Ureaplasma-driven neuroinflammation in neonates: assembling the puzzle pieces. Neonatology.

[CR42] Stoll BJ, Hansen NI, Bell EF, Walsh MC, Carlo WA, Shankaran S (2015). Trends in care practices, morbidity, and mortality of extremely preterm neonates, 1993–2012. JAMA.

[CR43] Sweeney EL, Dando SJ, Kallapur SG, Knox CL (2017). The human ureaplasma species as causative agents of chorioamnionitis. Clin Microbiol Rev.

[CR45] Viscardi RM (2014). Ureaplasma species: role in neonatal morbidities and outcomes. Arch Dis Child Fetal Neonatal Ed.

[CR48] Viscardi RM, Manimtim WM, Sun CC, Duffy L, Cassell GH (2002). Lung pathology in premature infants with Ureaplasma urealyticum infection. Pediatr Dev Pathol.

[CR47] Viscardi R, Manimtim W, He JR, Hasday JD, Sun CC, Joyce B (2006). Disordered pulmonary myofibroblast distribution and elastin expression in preterm infants with Ureaplasma urealyticum pneumonitis. Pediatr Dev Pathol.

[CR46] Viscardi RM, Hashmi N, Gross GW, Sun CC, Rodriguez A, Fairchild KD (2008). Incidence of invasive ureaplasma in VLBW infants: relationship to severe intraventricular hemorrhage. J Perinatol.

[CR49] Waites KB, Katz B, Schelonka RL (2005). Mycoplasmas and ureaplasmas as neonatal pathogens. Clin Microbiol Rev..

[CR50] Wang K, Wang H, Lou W, Ma L, Li Y, Zhang N (2018). IP-10 promotes blood-brain barrier damage by inducing tumor necrosis factor alpha production in japanese encephalitis. Front Immunol.

[CR51] Wevers NR, de Vries HE (2016). Morphogens and blood-brain barrier function in health and disease. Tissue Barriers..

[CR52] Williams JL, Holman DW, Klein RS (2014). Chemokines in the balance: maintenance of homeostasis and protection at CNS barriers. Front Cell Neurosci.

